# Differential Activation of a Mouse Estrogen Receptor **β** Isoform (mER**β**2) with Endocrine-Disrupting Chemicals (EDCs)

**DOI:** 10.1289/EHP396

**Published:** 2016-09-16

**Authors:** Lauren J. Donoghue, Thomas I. Neufeld, Yin Li, Yukitomo Arao, Laurel A. Coons, Kenneth S. Korach

**Affiliations:** Receptor Biology Section, Reproductive and Developmental Biology Laboratory, Division of Intramural Research, National Institute of Environmental Health Sciences, National Institutes of Health, Department of Health and Human Services, Research Triangle Park, North Carolina, USA

## Abstract

**Background:**

Endocrine-disrupting chemicals (EDCs) are suspected of altering estrogenic signaling through estrogen receptor (ER) α or β (mERβ1 in mice). Several EDC effects have been reported in animal studies and extrapolated to human studies. Unlike humans, rodents express a novel isoform of ERβ (mERβ2) with a modified ligand-binding domain sequence. EDC activity through this isoform remains uncharacterized.

**Objectives:**

We identified the expression pattern of mERβ2 in mouse tissues and assessed the estrogenic activity of EDCs through mERβ2.

**Methods:**

mERβ2 mRNA expression was measured in mouse tissues. HepG2 cells were used to assess the transactivation activity of mERβ isoforms with EDCs and ER co-activators. 293A cells transiently transfected with mER isoforms were used to detect EDC-mediated changes in endogenous ER target gene expression.

**Results:**

Expression of mERβ2 mRNA was detected in mouse reproductive tissues (ovary, testis, and prostate) and lung and colon tissues from both female and male mice. Five (E2, DES, DPN, BPAF, Coum, 1-BP) of 16 compounds tested by reporter assay had estrogenic activity through mERβ2. mERβ2 had a compound-specific negative effect on ERβ/ligand-mediated activity and ER target genes when co-expressed with mERβ1. mERβ2 recruited coactivators SRC2 or SRC3 in the presence of EDCs, but showed less recruitment than mERβ1.

**Conclusion:**

mERβ2 showed weaker estrogenic activity than mERβ1 in our *in vitro* system, and can dampen mERβ1 activity. *In vivo* models of EDC activity and ER-mediated toxicity should consider the role of mERβ2, as rodent tissue responses involving mERβ2 may not be reproduced in human biology.

**Citation:**

Donoghue LJ, Neufeld TI, Li Y, Arao Y, Coons LA, Korach KS. 2017. Differential activation of a mouse estrogen receptor β isoform (mERβ2) with endocrine-disrupting chemicals (EDCs). Environ Health Perspect 125:634–642; http://dx.doi.org/10.1289/EHP396

## Introduction

Estrogen receptors (ERs) mediate critical physiological events in many organ systems including the endocrine and reproductive systems (Henley and Korach 2010; McDonnell and Norris 2002; Nilsson et al. 2001). Both ERα and ERβ are members of the family of nuclear receptors (NRs) and have distinct functional domains that include ligand and DNA-binding regions. ERα and ERβ act as ligand-inducible transcription factors (TFs) upon binding estradiol (E_2_), the primary endogenous ER ligand (Hall and McDonnell 2005). ERβ is highly expressed in ovarian granulosa cells where it is required for effective ovulation (Couse et al. 2005; Rodriguez et al. 2010). Additional studies have suggested ERβ has a role in prostate cancer (Antal et al. 2012; Hartman et al. 2012). Together, these processes highlight the importance of regulated ERβ signaling.

ERs are evolutionarily conserved between species, including between humans and rodents (Lewandowski et al. 2002). In the 1990s, several studies reported the discovery of a splice-variant of ERβ in rats and mice (Chu and Fuller 1997; Hanstein et al. 1999; Maruyama et al. 1998; Petersen et al. 1998). This variant, termed mERβ2 in the mouse, contains an 18-amino acid insert in the ERβ ligand-binding domain (LBD). This corresponds with a 54 nucleotide insert located between exon 5 and exon 6 of the *Esr2* gene. A splice accepting site in this region of the mouse *Esr2* gene allows for the altered inclusion of this segment, whereas this splicing site does not exist in the human *ESR2* gene. A transcript with a similar insert has not been detected in human cells (Lu et al. 1998).

Upon activation, ERs are localized to the nucleus where they direct transcription by binding estrogen-response element (ERE) sequences located in the regulatory regions of target genes. ERs can also interact with other TFs on sites such as AP-1 or Sp1 to regulate gene expression (Hall and McDonnell 2005; O’Lone et al. 2004). Their interactions with the RNA polymerase II complex and the chromatin environment that surrounds the genes depend upon and are modified by co-regulators (Tsai and O’Malley 1994). The steroid receptor coactivator (SRC) family in mice contains three homologous members, SRC-1/NCoA-1, SRC-2/NCoA-2, and SRC-3/NCoA-3. Each member of the SRC family is able to potentiate the transcriptional activities of NRs, including ERs (Lonard and O’Malley 2007, 2012).

Endocrine-disrupting chemicals (EDCs) are exogenous compounds that interfere with homeostasis by disrupting endogenous hormone synthesis or signaling, including the binding to ER or other receptors. The possible health effects of EDCs are of growing scientific concern (Diamanti-Kandarakis et al. 2009). As a class, EDCs are ubiquitous in the environment, and prominent EDCs such as bisphenol A (BPA) and phytoestrogens have been detected in ecosystems and human serum at high concentrations (Berman et al. 2013; Hormann et al. 2014). ERs are classical targets for possible EDC-mediated toxicity (Diamanti-Kandarakis et al. 2009). Studies including *in vitro* and *in vivo* assays have provided evidence for such toxicity, including embryo implantation defects, developmental effects and changes in behavior and memory following EDC exposure (Jefferson et al. 2012; Sobolewski et al. 2014). Studies have classified many synthetic and natural chemicals as EDCs (Henley and Korach 2010). Synthetic EDCs such as BPA are used in a variety of industries and products. BPA can impede the activity of endogenous estrogens by disrupting the proper activity of ERs in a diverse set of target tissues (Wetherill et al. 2007). Some natural phytoestrogens such as daidzein (Dai) and genistein (Gen) exist in foods derived from plants, especially soy-based foods (Dang 2009). Actions of EDCs and toxicity effects in experimental studies have been extrapolated to humans and have been used for proposed regulatory guidelines (Shelnutt et al. 2013).

Previously, we used human HepG2 (hepatocellular carcinoma), HeLa (cervical carcinoma), and Ishikawa (endometrial carcinoma) cell lines to analyze the ER-mediated estrogenic effects of a set of EDCs (Li et al. 2012, 2013). Our studies demonstrated the mechanistic importance of chemical structure similarities and cell type and gene promoter specificity when evaluating the potential activities of EDCs, including synthetic EDCs such as BPA and its analogs, and natural EDCs such as Dai and Gen (Li et al. 2013).

mERβ2 has reduced activity with E_2_ compared with the primary form, mERβ1, yet the activity of this isoform with EDCs is unknown (Zhao et al. 2005). Although an ERβ2 isoform with a similar insert has not been identified in human tissues, rodent models are research assets in assessing the physiological effects and toxicity of EDCs. In the present study, we characterized mERβ2 expression in mouse tissues to evaluate if its expression may have any potential to impact reported toxicity. Using the HepG2 cell line as an *in vitro* model, we tested the estrogenic activity of 16 EDC compounds, including endogenous hormones and EDCs, by luciferase reporter assay. We mechanistically investigated mERβ2’s potential for exhibiting a reducing or dominant-negative effect on the ERβ/ligand-mediated activity of mERβ1, as well as mERβ isoform-specific recruitment of SRCs, to evaluate the impact of mERβ2 on EDC activity and toxicity assessment. In addition, we determined the activity as well as dominant-negative effects of mERβ2 on expression of endogenous ER target genes such as *GREB1* (gene regulation by estrogen in breast cancer 1) and *PGR* (progesterone).

## Materials and Methods

### Reagents

The chemicals used in this study are as follows: 17β-estradiol (E_2_) and diethylstilbestrol (DES) were purchased from Sigma-Aldrich (St. Louis, MO); the endogenous hormone metabolites 3β-androstanediol/5α-androstane-3β,17β-diol (3β-diol), and 5-androstenediol/androst-5-ene-3β,17β-diol (Δ^5^-diol) were purchased from Research Plus Steroid Laboratories, Inc. (Denville, NJ); 2,3-*bis*(4-hydroxyphenyl)-propionitrile (DPN) was purchased from TOCRIS Bioscience (Avonmouth, Bristol, UK); bisphenol A (BPA), bisphenol AF (BPAF), 2-2-*bis*(p-hydroxyphenyl)-1,1,1-trichloroethane (HPTE), 4-*n*-nonylphenol (4-*n*-NP), diadzein (Dai), genistein (Gen), kaempferol (Kaem), apigenin (Api), coumestrol (Coum), endosulfan (Endo), kepone (Kep), and 1-bromopropane (1-BP) were obtained from the Midwest Research Institute (Kansas City, MO) through a contract with the National Toxicology Program and are previously described (Li et al. 2013). The chemical structures are shown in Table S1. Chemical Abstracts Services Registry Numbers (CAS No.) and the sources are summarized in Table S2. All chemicals were dissolved in dimethyl sulfoxide (DMSO).

### Plasmids

**Figure 1:**
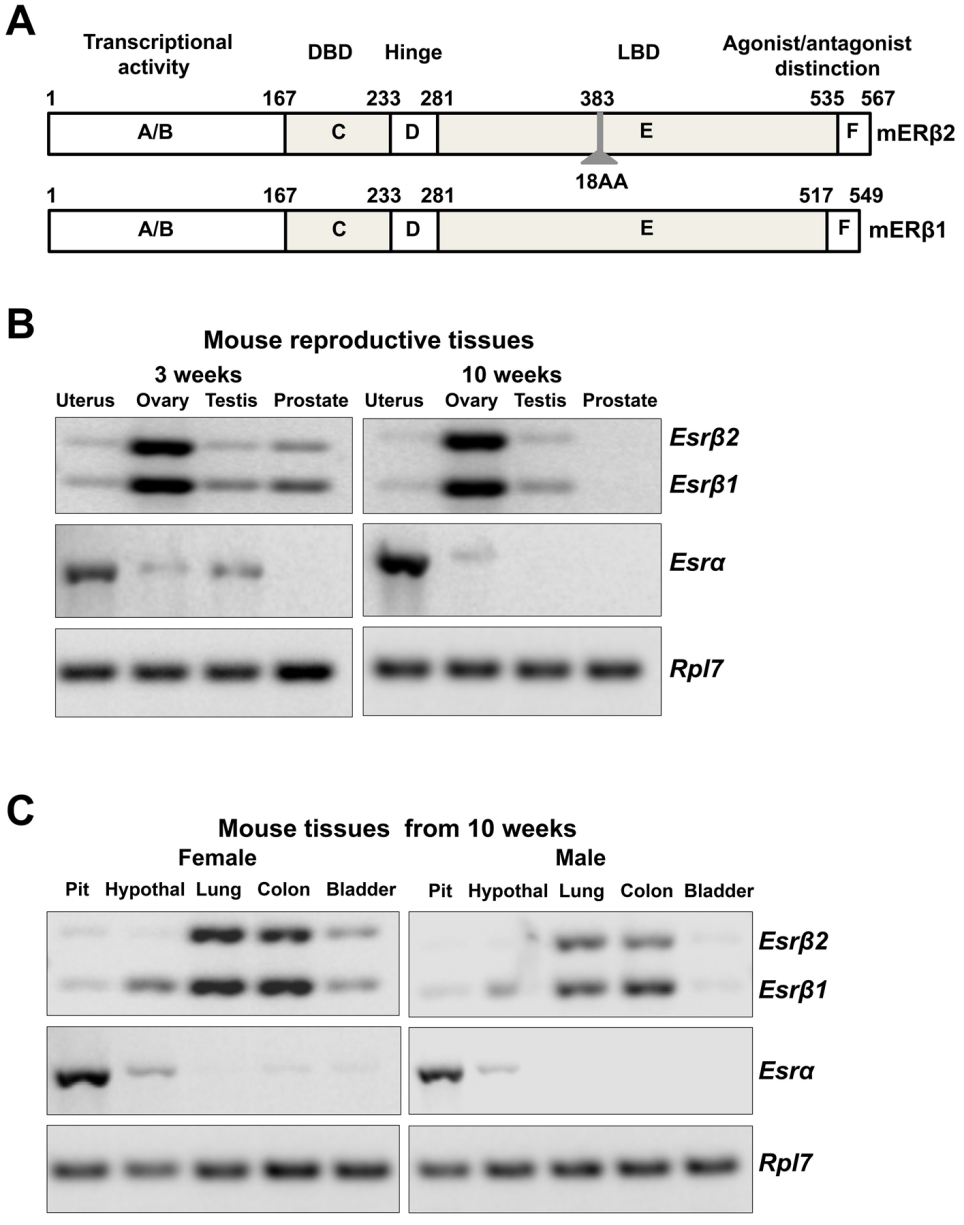
Gene expression levels of mERβ isoforms and mERα in mouse tissues. (*A*) A structural diagrammatic comparison of mERβ2 and mERβ1 protein. (*B*) Gene expression in mouse reproductive tissues. mRNA was isolated from uterus, ovary, testis, or prostate from 3- or 10-week-old C57/BL6 mice. Expression of mERβ1, mERβ2, and mERα mRNA was detected by RT-PCR and presented in a gel electrophoresis. mRNA level of ribosomal protein L7 (*Rpl7*) was used as a loading control. (*C*) Gene expression in female and male mouse tissues. mRNA was isolated from pituitary, hypothalamus, lung, colon, or bladder from 10-week-old female and male mice. Detection of mRNA levels is as described in (*B*).

The mouse ERβ2 expression plasmid (pcDNA3-mERβ2, mERβ2) was generated as follows: the cDNA fragment of mouse ovarian ERβ was amplified by polymerase chain reaction (PCR) using the following primer set, mERβ-C-ter_5´; 5´-CAA GTG TTA CGA AGT AGG AAT GGT CAA GTG TGG-3´ and mERβ-C-ter_3´; 5´-TCT CTG CTT CCT GGC TTG CGG TAG C-3´. The amplified fragment was cloned into pCR2.1 using TA Cloning kit (Invitorogen, Carlsbad, CA) and sequenced. Next, the KpnI-PstI fragment from the pCR2.1-mERβ-C-ter-L was inserted into the KpnI-PstI sites of pBluescript-mERβ-C-terminal plasmid (the KpnI-XbaI fragment of pcDNA3-WTmERβ was subcloned to the pBluescript plasmid). Finally, the KpnI-XbaI fragment of pBluescript-mERβ-C-terminal-L plasmid was subcloned to the KpnI-XbaI sites of pcDNA3-WTmERβ. The mouse ERβ1 expression plasmid pcDNA3-WTmERβ (mERβ1) has been previously described (Li et al. 2013). A structural diagrammatic comparison of mERβ2 and mERβ1 is shown in Figure 1A. The luciferase reporter plasmid pGL3/3xERE-TATA-Luc (3xERE Luc) contained three repeats of the vitellogenin consensus ERE (Burns et al. 2011). The expression vector pcDNA was purchased from Invitrogen (Carlsbad, CA). The renilla luciferase expression plasmid pRL-TK was purchased from Promega (Madison, WI). The coactivator expression plasmids, pcDNA/SRC1 (SRC1), pcDNA/SRC2 (SRC2), and pcDNA/SRC3 (SRC3) were gifts from Dr. Donald McDonnell (Duke University).

### Cell Lines and Tissue Culture

The HepG2 human hepatocellular carcinoma cell line was purchased from ATCC (Manassas, VA). The 293A human kidney epithelial cell line was purchased from Invitrogen (Carlsbad, CA). Cells were maintained in phenol red–free minimum essential medium (MEM) (Gibco™) for HepG2 culture or phenol red–free DMEM medium (Gibco™) for 293A culture supplemented with 10% fetal bovine serum (FBS; Gemini Bio Products, West Sacramento, CA) 5 mM L-glutamine (Invitrogen, Carlsbad, CA), and streptomycin (Gibco™).

### Mice and Tissue Collection

All animal studies were conducted in accordance with the NIH Guide for the Care and Use of Laboratory Animals and approved by the National Institute of Environmental Health Sciences (NIEHS) Animal Care and Use Committee. Animals were treated humanely and with regard for alleviation of suffering. Tissues were collected from both male and female C57/BL6 mice purchased from Charles River Laboratories (Wilmington, MA) and shipped to the NIEHS. Tissues were collected at 3 and 10 weeks of age, frozen on dry ice, and stored at –80°C until RNA extraction.

### RNA Extraction from Mouse Tissues and RT-PCR Conditions

RNA was isolated with Trizol reagent (Invitrogen, Carlsbad, CA) and reverse transcribed with SuperScriptII (Invitrogen, Carlsbad, CA) according to the manufacturer’s protocol. ERβ (*Esr2*), ERα (*Esr1*) transcripts were amplified by PCR in a reaction mixture containing RedTaq^®^ Ready Mix (Sigma-Aldrich, St. Louis, MO). The primer pairs for *Esr2* were (forward; 5´-GCT​CAT​CTT​TGC​TCC​AGA​CCT​CGT​TCT-3´ and reverse; 5´-GGT​ACA​TAC​TGG​AGT​TGA​GGA​GAA​TCA​TGG​C-3´) and *Esr1* (forward; 5´-AAG​CTG​GCC​TGA​CTC​TGC​AG-3´ and reverse; 5´-TGT​TGT​AGA​GAT​GCT​CCA​TGC​C-3´). PCR for ribosomal protein L7 (*Rpl7*) served as a normalization gene, with primer pairs (forward; 5´-AGC​TGG​CCT​TTG​TCA​TCA​GAA-3´ and reverse; 5´-GAC​GAA​GGA​GCT​GCA​GAA​CCT-3´). The PCR conditions for all transcripts were as follows: 32 cycles of 95°C 30 sec, 65°C 30 sec, 72°C 1 min. The amplicons of *Esr2* isoform 2 (mERβ2) and *Esr2* isoform 1 (mERβ1) were 230 bp and 176 bp, respectively. PCR products were separated by gel electrophoresis in 2% agarose and visualized with GelRed nucleic acid stain (Biotium, Hayward, CA).

### Transient Transfection, Cell Treatment, and Luciferase Assay

HepG2 cells were seeded at 1.2 × 10^–5^ cells/well in 24-well plates overnight in medium with 10% charcoal- and dextran-stripped FBS (Thermo Scientific, Waltham, MA) substituted for FBS. The following morning, cells were changed to fresh medium and transfected for 6 hr using Effectene transfection reagent (QIAGEN, Valencia, CA) according to the manufacturer’s protocol. A total of 0.5 μg (mERβ2, mERβ1 or both) or 0.7 μg (mERβ2 or mERβ1 with SRCs) were transfected in cells (details of the DNA components are summarized in Table S3). Six to eight hours after transfection, cells were changed to fresh medium and kept in culture for 20–24 hr and then treated with DMSO vehicle (control, final concentration ≤ 0.05%), or from 10^–12^ M to 10^–6^ M of the chemicals tested. After 18 hr, cell lysis and luciferase assays were performed using the Dual Luciferase Reporter Assay System (Promega, Madison, WI). Transfection efficiency was normalized by renilla luciferase using pRL-TK plasmid. All experiments were repeated in triplicate in at least three independent trials.

### Endogenous Gene Expression Analysis Following Transient Transfection and Treatment

293A cells were seeded in 6-well plates and then were transiently transfected with 1 μg mERβ1, mERβ2, or both isoforms (1:1 ratio) for 8 hr. After starving cells overnight, cells were treated with vehicle (control), 10^–8^ M E2 (for mERβ1), 10^–7^ M E2 (for mERβ2) or 10^–6^ M DPN, BPA, HPTE or Gen for 18 hr. Total RNA was extracted by using the RNeasy Mini Kit (Qiagen, Valencia, CA) and reverse transcribed with SuperScriptII™ (Invitrogen, Carlsbad, CA) according to the manufacturer’s protocol. The mRNA levels of ER target genes were measured using SYBR green assays (Applied Biosystems, Carlsbad, CA). The sequences of primers used in real-time PCR were as follows: for human *GREB1* (NM_014668): the forward primer 5´-CAA​AGA​ATA​ACC​TGT​TGG​CCC​-3´, reverse primer 5´-GAC​ATG​CCT​GCG​CTC​TCA​TAC​-3´; human *PR* (NM_000926.4): the forward primer 5´-GAC​GTG​GAG​GGC​GCA​TAT​-3´, reverse primer 5´-GCA​GTC​CGC​TGT​CCT​TTT​CT-3´. Cycle threshold (Ct) values were obtained using the ABI PRISM^®^ 7900 Sequence Detection System and analysis software (Applied Biosystems, Foster City, CA). Each sample was quantified against its β-actin transcript content: using forward primer 5´-GAC​AGG​ATG​CAG​AAG​GAG​ATC​AC-3´ and reverse primer 5´-GCT​TCA​TAC​TCC​AGC​AGG-3´. The experiments were repeated three times and results are presented as fold increase calculated relative to the vehicle (control) for each group.

### Statistical Analysis

**Figure 2:**
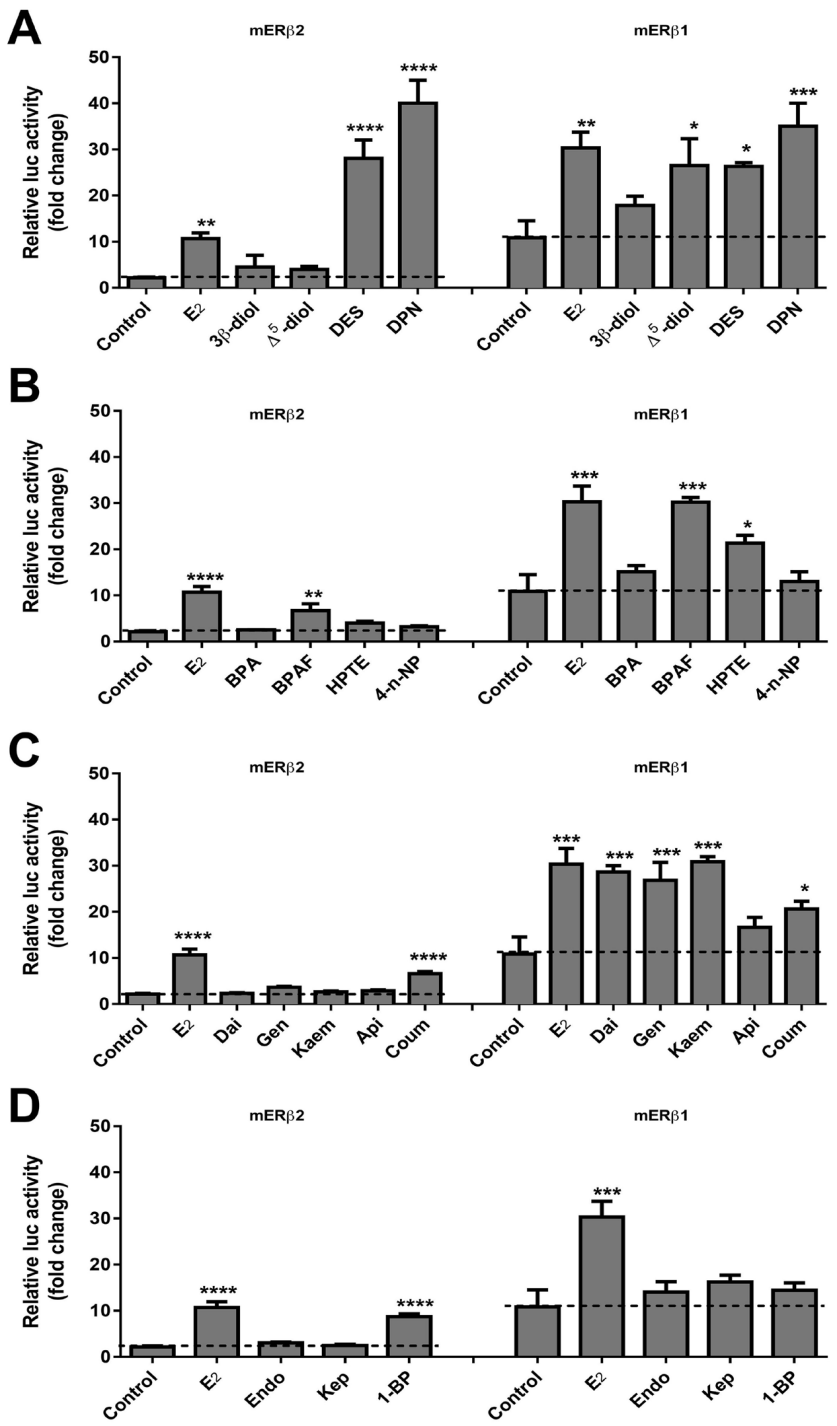
The mERβ2 isoform has reduced activation with EDCs compared to mERβ1. (*A*) Estrogenic activation by endogenous hormones (E_2_, 3β-diol, or ∆^5^-diol) and pharmaceutical chemicals (DES or DPN). (*B*) Activity by the Group 1 EDCs (BPA, BPAF, HPTE, 4-*n*-NP). (*C*) Activity by the Group 2 EDCs (Dai, Gen, Kaem, Api, Coum). (*D*) Activity by the Group 3 EDCs (Endo, Kep, 1-BP). HepG2 cells were transfected with a 3xERE-luc reporter plasmid, pRL-TK transfection normalization plasmid, and expression plasmid for mERβ1 or mERβ2, allowed to recover for 18 hr, and treated with E_2_ at 10^–8^ M or compounds at 10^–7^ M for 18 hr. The luciferase activity is presented as relative activity compared with the vehicle treated cells transfected with empty pcDNA3 plasmid. The relative activity is represented as the mean ± SEM. Significant difference is based on comparison to mERβ2 control (for mERβ2 treatments) or mERβ1 control (for mERβ1 treatments). Assays were run in triplicate and data replicated over at least three independent experiments. *p < 0.05. **p < 0.01. ***p < 0.001. ****p < 0.0001.

**Figure 3:**
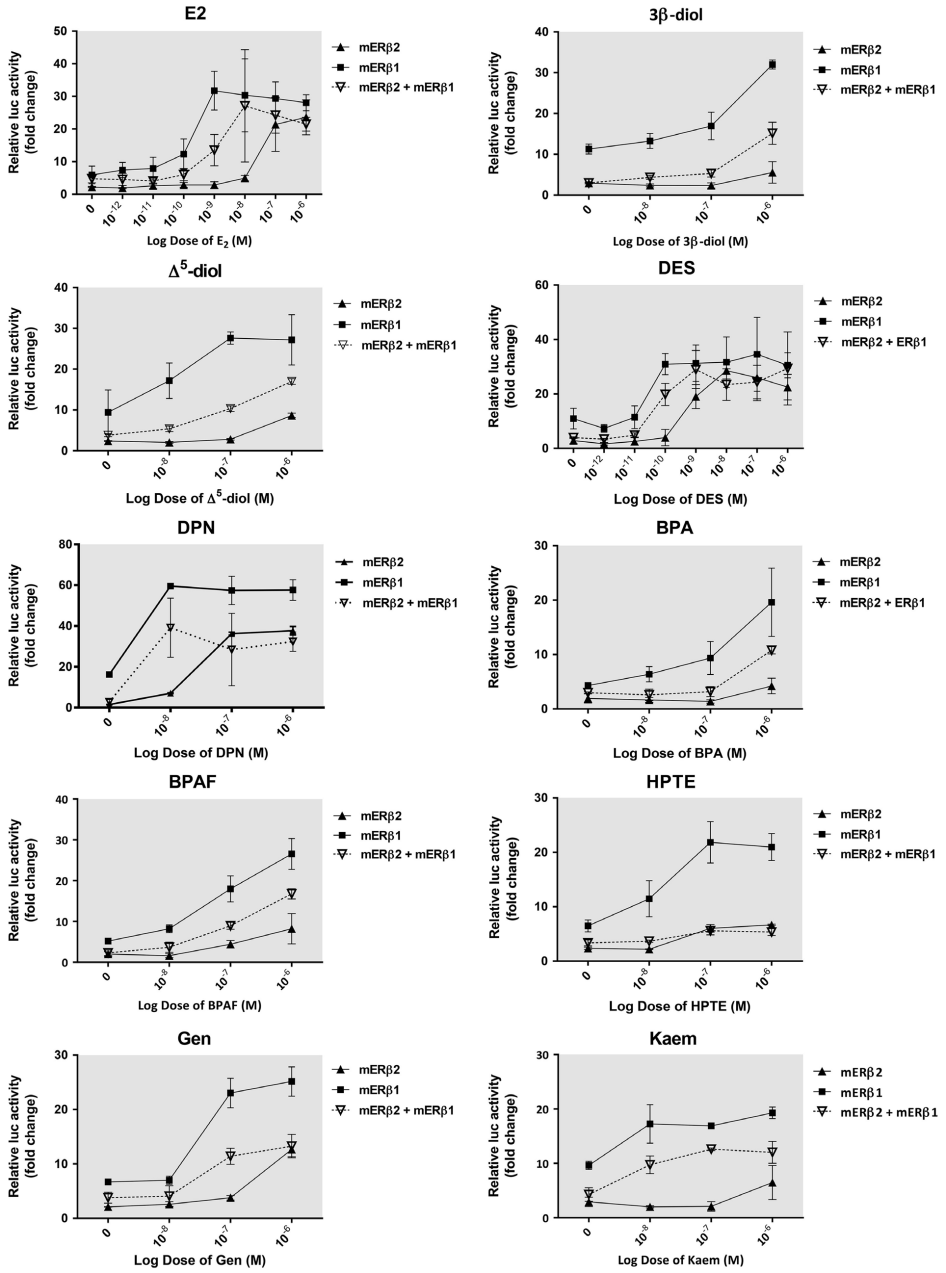
Co-expression of mERβ1 and mERβ2 attenuated activity compared to mERβ1 following EDC treatment. HepG2 cells were transfected with the 3xERE-luc reporter plasmid, pRL-TK transfection normalization plasmid, and the expression plasmid for mERβ1, mERβ2, or both mERβ1 and mERβ2, allowed to recover for 18 hr, and then treated with E2, 3β-diol, Δ5-diol, DES, DPN, BPA, BPAF, HPTE, Gen, and Kaem across increasing concentrations for 18 hr. Luciferase activity is shown as mean fold change to vehicle treated cells transfected with empty pcDNA3 plasmid. Assays were run in triplicate and data replicated over at least three independent experiments. Error bars on each data point represent 95% confidence intervals around that value.

**Figure 4:**
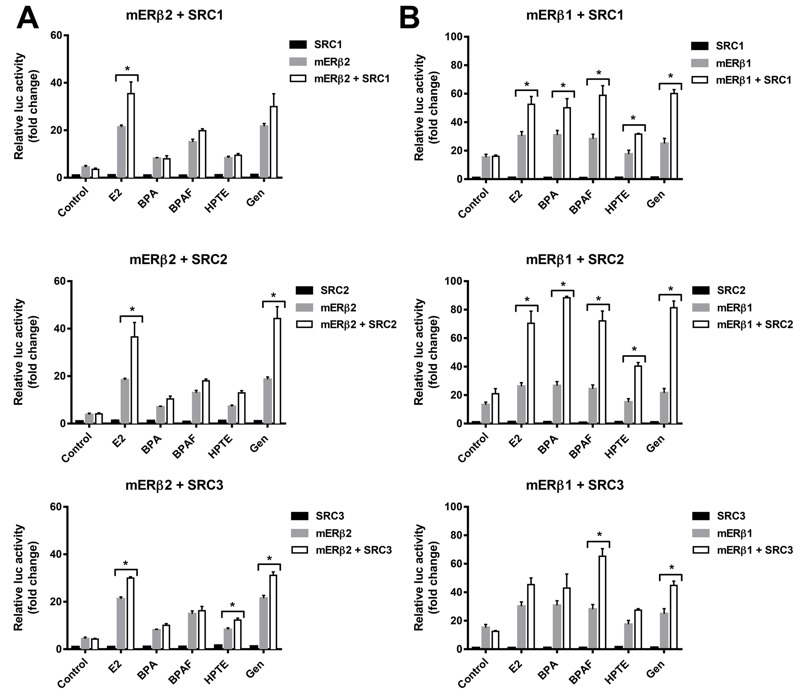
ER co-activator SRC family proteins have greater effect on mERβ1 response than on mERβ2 response. (*A*) Estrogenic activation on mERβ2 with SRCs. (*B*) Estrogenic activation on mERβ1 with SRCs. Cells were transfected with a 3xERE-luc reporter plasmid, pRL-TK plasmid, mERβ2 or mERβ1 expression plasmid, and expression plasmids for SRC1, SRC2, or SRC3. After 18 hr, transfected cells were treated with E_2_ at 10^–7^ M (for mERβ2) or 10^–8^ M (for mERβ1), or BPA, BPAF, HPTE, or Gen at 10^–6^ M. The luciferase activity is represented as relative activity compared with the vehicle treated cells transfected with SRC expression plasmid and mERβ1 or mERβ2 expression plasmid. The relative activity is represented as the mean ± SEM. Significant difference was analyzed between a treatment with mERβ plasmid alone, and the corresponding treatment with mERβ plasmid and SRC plasmid. Assays were run in triplicate and data replicated over at least three independent experiments. *p < 0.05.

**Figure 5:**
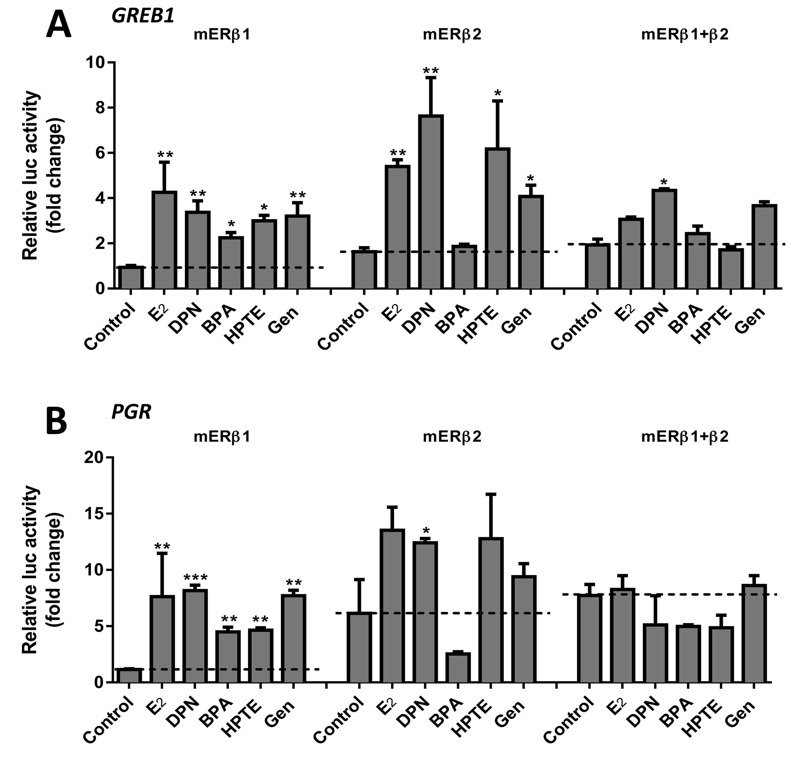
The effects of EDCs on the ER target gene expression of (*A*) *GREB1* and (*B*) *PGR* in 293A cells. Cells were transiently transfected with mERβ1, mERβ2 or both isoforms for 8 hr and then starved overnight. Cells were treated with the vehicle (control), 10^–8^ M E2 (for mERβ1), 10^–7^ M E2 (for mERβ2), or 10^–6^ M DPN, BPA, HPTE, or Gen for 18 hr. Total RNA was isolated, and mRNA levels of *GREB1* and *PGR* were quantified by real time-PCR. Target gene expression was normalized to β*-actin* gene expression. Data shown is representative of triplicates as fold increase calculated relative to the vehicle (control) ± SEM. *p < 0.05, **p < 0.01, ***p < 0.001 compared with the vehicle (control) for each group.

All analyses were performed using GraphPad Prism (version 6.0; GraphPad Software Inc., La Jolla, CA). For data in Figure 2 (and also in Figure 5), we used two-way ANOVA with Dunnett’s correction for multiple comparisons to determine statistical signifcance for each group compared with the control group: The differences are based on comparing the mERβ2 control (for mERβ2 treatments) or the mERβ1 control (for mERβ1 treatments). For Figure 3, error bars for each data point represent 95% confidence intervals around that value. For Figure 4, multiple *t*-tests (with Sidak-Bonferroni correction for multiple comparisons) were used to analyze the differences between a treatment with mERβ plasmid alone and the corresponding treatment with mERβ plasmid and SRC plasmid.

## Results

### MER *β* 2 mRNA Expression Level Is Dependent on Tissue Type and Maturity

To characterize the tissue types where estrogenic signaling may be mERβ2 dependent on the age-dependent expression of mERβ2, we extracted mRNA from mouse tissues at 3 or 10 weeks of age. We amplified mERβ2, mERβ1, and mERα transcripts by RT-PCR (Figure 1B,C). For the reproductive tissues examined, the mouse ovary had the highest detected level of both of mERβ2 and mERβ1 at both ages. The mouse testis had lower expression of both mERβ isoforms at either age. The mouse uterus and prostate expressed both isoforms of mERβ at 3 weeks of age, but all forms were undetectable by 10 weeks of age (Figure 1B). The lung and colon in both the female and male tissues at 10 weeks of age had the second highest levels of mERβ2 detected in any tissue, followed by the bladder, in which both forms of mERβ were only detected in female tissues (Figure 1C). Detectable expression of only mERβ1 and mERα, but not mERβ2, was found in female hypothalamus. No mRNA from either mERβ isoform was detected from the thymus, heart, stomach, kidney, or liver in female or male tissues at 10 weeks of age (data not shown). As expected, the mouse uterus and pituitary had the highest expression of mouse ERα. These data indicate that mERβ2 is co-expressed with the primary mouse ERβ isoform, mERβ1, in a sex dependent tissue-selective manner.

### EDCs in Transactivation Activity Screening Selectively Activate mER *β* 2

We assessed the ERE-mediated transcriptional response of mERβ2 and mERβ1 upon treatment with a panel of 16 compounds, including hormones, pharmaceutical chemicals, and synthetic and natural EDCs. First, we constructed the mERβ2 expression plasmid using the mouse ovary tissue as a template and then confirmed that mERβ2 isoform had an additional 18 amino acids in the LBD as reported (Hanstein et al. 1999). ER-negative HepG2 cells were transfected with receptor expression plasmid and the 3xERE reporter plasmid. All compounds were evaluated at 10^–7^ M as performed in our previous studies, except for E_2_ for mERβ1, which was evaluated at the more physiologically relevant concentration of 10^–8^ M (Li et al. 2013). The receptor activation relative to vehicle-treated control cells is shown in Figure 2. The basal activity of mERβ2 is dramatically lower than mERβ1. E_2_, DPN, and DES significantly induced both mERβ2- and mERβ1- mediated transcriptional activation, while DES significantly induced mERβ2-mediated activation only. Treatment with 3β-diol did not activate either mERβ isoform at the tested concentration (Figure 2A), while Δ^5^-diol activated only mERβ1. Of the BPA analogs, BPAF significantly activated both mERβ isoform-mediated responses. HPTE selectively activated mERβ1, but not mERβ2. BPA and 4-*n*-NP were not active on either mERβ isoform (Figure 2B). Of the natural phytoestrogens, only Coum showed mERβ2-mediated activity; Dai, Gen, Kaem, and Coum activated mERβ1; Api did not activate either of the mERβs (Figure 2C). In addition, 1-BP showed significant activation for only mERβ2 (Figure 2D). These results demonstrate that EDCs can induce mERβ ERE-mediated transactivation in a compound and isoform-specific manner.

### MER *β* 2 Has Dose-Dependent Transcriptional Activity and a Reductive Regulatory Role for mER *β* 1 Activity

To further evaluate the potential functionality of mERβ2 and its apparent reduced estrogenic activity, 10 compounds were selected for analysis of activation across a range of concentrations based on their activities in Figure 2: E_2_, 3β-diol, Δ^5^-diol, DES, DPN, BPA, BPAF, HPTE, Gen, and Kaem. Cells were transiently transfected with mERβ2, mERβ1, or both isoforms in HepG2 cells. A 1:1 ratio of mERβ2 and mERβ1 expression plasmids was chosen for co-transfected cells to mimic the endogenous expression levels observed in the mouse ovary (Figure 1A). E_2_ and DES were evaluated from 10^–12^ M to 10^–6^ M and 3β-diol, Δ^5^-diol, DPN, BPA, BPAF, HPTE, Gen, and Kaem were evaluated from 10^–8^ to 10^–6^ M (Figure 3). Co-expression of mERβ2 and mERβ1 in cells treated with all chemicals showed activation below that of mERβ1 and above that of mERβ2 alone, indicating an attenuation of the mERβ1 activity. Across all doses of E2, DES and DPN treatments, cells co-expressing the isoforms exhibited significantly less activation than cells singly-expressing mERβ1; 3β-diol and Δ^5^-diol treatment resulted in activation with co-transfected mERβ1 and mERβ2 that was ~ 30% of the value found in mERβ1-expressing cells. BPA, BPAF, and Gen treatment resulted in activation with mERβ1 and mERβ2 that was ~ 50% of the value in mERβ-expressing cells. In contrast to all other treatments, treatment with HPTE resulted in co-expressed mERβ1 and mERβ2 having no activation higher than cells singly expressing mERβ2 at any dose. In addition, co-expression of mERβ2 and mERα in cells treated with 10^–8^ M of EDCs also resulted in attenuation of activation below that of mERα and above that of mERβ2 when expressed alone (see Figure S1). These data indicate that mERβ2 can reduce the mERβ1 activity that is ligand-dependent across varying ligand concentrations.

### SRC ER Coactivators Have Greater Effects on mER *β* 1 Responses than on mER *β* 2 Responses

To determine if the reducing and dominant-negative activity of mERβ2 was in part due to differential and ligand-specific co-regulator recruitment, we co-transfected the mERβ2 or mERβ1expression plasmid with SRC1, SRC2, or SRC3 expression plasmid in HepG2 cells and then treated with 10^–8^ M E_2_ (mERβ1), 10^–7^ M E_2_ (mERβ2), or 10^–6^ M BPA, BPAF, HPTE or Gen (Figure 4). In the presence of E_2_, SRC1, SRC2 and SRC3 significantly co-activated ERE-mediated activity for mERβ1 and mERβ2. In contrast, with EDC treatment, mERβ2 showed reduced activation and SRC-selective activation. SRC2 and SRC3, but not SRC1, co-activated mERβ2 after Gen treatment and none of the three SRCs co-activated with mERβ2 in the presence of the other EDCs (BPA, BPAF or HPTE). SRC3 showed weak co-activation of mERβ2 with HPTE treatment (Figure 4A). In comparison, SRC1 and SRC2 significantly increased the mERβ1 ERE-mediated activity in the presences of all five compounds (E_2_, BPA, BPAF, HPTE or Gen). SRC3 co-activated only with mERβ1 in the presence of BPAF and Gen, but not E_2_ (Figure 4B). To further explore the isoform-specific recruitment of coactivators, we co-transfected both of mERβ2 and mERβ1 with SRC2. With E_2_ treatment, the presence of mERβ2 reduced the mERβ1-mediated activation without SRC2, but there was an induction with SRC2 transfection (see Figure S2). However, with DPN or HTPE treatment, SRC2 did not change the mERβ2 and mERβ1 co-activation (see Figure S2). These data demonstrate that the mERβ isoforms differentially recruited SRC coactivators in an EDC-specific manner and that coactivators have greater effects on mERβ1-mediated responses than mERβ2-mediated responses.

### MER *β* 2 Has a Reductive Effect on EDC-Induced mER *β* 1 Gene Expression

To investigate the apparent effect by mERβ2 on regulation of endogenous ER target genes, 293A cells were transiently transfected with mERβ1 or mERβ2, or both of isoforms. Using real time-PCR, we measured expression of *GREB1* and *PGR* after treatment with 10^–8^ M E_2_ (for mERβ1 and mERβ1 + mERβ2), 10^–7^ M E_2_ (for mERβ2), or 10^–6^ M DPN, BPA, HTPE or Gen. Fold changes in gene expression, relative to vehicle (control), are shown in Figure 5. For mERβ1, all compounds (E_2_, DPN, BPA, HTPE, and Gen) significantly induced both *GREB1* and *PGR* endogenous gene expression. For mERβ2, E_2_, DPN, HPTE, and Gen significantly induced *GREB1*, while only DPN induced the *PGR* expression. *PGR* was weakly stimulated by E_2_ and HPTE but more significantly by DPN. However, BPA did not induce expression of either gene. In cells co-transfected with both isoforms, no compound had significant induction of *GREB1* or *PGR* gene expression, with the exception of weak induction of *GREB1* by only DPN. These data indicate that mERβ2 has an attenuating effect on mERβ1-mediated induction of ER target genes by EDCs.

## Discussion

Investigating the physiological health effects of EDCs using mouse models requires an extensive understanding of mechanistic estrogenic signaling in the mouse that can be extrapolated to human clinical findings. ERβ has been implicated in adverse phenotypes and reproductive processes following EDC exposure (Le and Belcher 2010; Pellegrini et al. 2014). There is an isoform of ERβ that is rodent specific and has not been identified in human samples (Lewandowski et al. 2002; Lu et al. 1998). In this study, we investigated the expression and activity of the mERβ2 isoform to evaluate its potential role in mediating the estrogenic and toxic effects of EDCs. This knowledge of mERβ2 activity is necessary for translational research studies and for interpretation of EDC-exposure data in animals toward predicted effects in the human population. EDCs that show no exposure effects in experimental animal studies due to the mERβ2’s attenuation of estrogen signaling could subsequently result in positive human clinical effects since the ERβ2 isoform is not present. Likewise, the magnitude of the effects from experimental studies may be lessened because of the presence and dampening of tissue-specific mERβ2 activity.

Previous studies have examined the tissue distribution of mERβ2 with inconsistent results (Maruyama et al. 1998; Petersen et al. 1998). Through analysis of mRNA expression of mERβ1 and mERβ2, we found that mERβ1 was present in all tissues where mERβ2 was expressed. These results were replicated in at least five individual mice. The ratio of mERβ1 expression to mERβ2 expression was dependent on tissue type. The ovary had an approximately 1:1 ratio of mERβ1 to mERβ2 expression. ERβ is known to have a critical role in the ovary to facilitate ovulation and follicle development (Binder et al. 2013; Couse et al. 2005; Deroo and Korach 2006). ERβ has been shown to play a role in ovarian response to gonadotropins during ovulation (Couse et al. 2005). Previous studies have investigated ovarian toxicity by a suite of EDCs (Patel et al. 2015) in mice, as well as the effect of BPA on expression of the mouse *Esr1* and *Esr2* genes (Berger et al. 2016). However, we are not aware of *in vivo* studies of EDC activity that specifically account for the reductive activity of mERβ2. The mERβ2 isoform was also found in other tissues including the prepubertal uterus, prostate, lung, colon, and bladder, at a lower expression ratio than in the ovary (1:2 or 1:3 ratio of mERβ1 to mERβ2 expression). This unequal distribution in comparison to mERβ1 in tissues suggests that mERβ2 may have a more significant effect for some organ systems than others.

Previous studies have reported that mERβ2 requires from 10-fold to 1,000-fold higher concentration of E_2_ to reach the activity of mERβ1 (Petersen et al. 1998; Zhao et al. 2005). In this study, mERβ2 had significantly reduced activation compared with mERβ1 from 10^–12^ M to 10^–6^ M. ER activation with EDC treatment has been shown to be cell type and reporter specific (Gaido et al. 1999; Li et al. 2013), and we believe these factors contribute to the differing conclusions in the literature regarding the dose of E_2_ to reach maximum mERβ2 activity. Furthermore, our findings reveal that mERβ2 has either reduced activation or no activation with a number of EDCs or endogenous ligands for mERβ1. For the EDC compounds that were tested for promoter activity with a 3xERE reporter, mERβ2 had ~ 15% to ~ 50% of the activation level of mERβ1. The weak activity of mERβ2 with EDCs could skew the profile of mERβ signaling such that it appears less active in rodents than in clinical or epidemiological analyses since humans have no such ERβ2 isoform present known to dampen the potential toxicity. mERβ2 had the greatest activation following BPAF, 1-BP, or Coum treatment. DES (a known potent estrogen) and DPN (an ERβ agonist) were exceptions and can activate mERβ2 to a greater level than mERβ1. These data suggest that mERβ2 may not be capable of inducing endogenous gene expression following treatment with select EDCs. This reduced activity is likely due to reduced ligand-binding affinity, which was previously reported for E_2_, DES, BPA, and Gen (Petersen et al. 1998; Zhao et al. 2005) and to reduced SRC recruitment with EDCs, which we report here. In this study, we found that following EDC treatment, mERβ2-induced endogenous gene expression was limited compared with that of mERβ1 and that only certain ER target genes, such as *GREB1* but not *PGR,* were induced. The lack of activation by mERβ2 following treatment with 3β-diol and Δ^5^-diol, reported as endogenous high-affinity ligands for mERβ (Kuiper et al. 1997; Miller et al. 2013), suggests that if mERβ2 does have a high-affinity endogenous ligand, it may be different than those reported for mERβ1. It therefore may be intended for a different physiological role. In Saijo et al. (2011), researchers observed reduced inflammation in microglia upon treatment with Δ^5^-diol, but not E_2_. Until a mERβ2-specific agonist is identified, target genes of mERβ2 and the ability of mERβ2 to shift the estrogen responsiveness of tissues cannot be evaluated (Saijo et al. 2011).

Our study is the first to investigate what may be considered a reductive regulatory role for mERβ2 on mERβ1 following EDC treatment. Consistent with a previous report of dominant negative regulation by ERβ2 in the rats (Hanstein et al. 1999), we found that in a 1:1 co-expression of mERβ2 and mERβ1 in HepG2 cells, which represents the levels found in ovarian granulosa cells, the transactivation activity of mERβ2 and mERβ1 is significantly reduced compared to that of mERβ1 alone. This pattern of activity is also observed in cells with 1:1 co-expression of mERα and mERβ2 (see Figure S1). After examining responses to ligands across a range of concentrations, this dominant negative activity was determined to be compound specific. For example, co-expression of mERβ2 and mERβ1 resulted in significantly less transactivation than in mERβ1expressing cells but more than in mERβ2 expressing cells upon treatment with BPA or BPAF, yet activity following HPTE treatment is not significantly different than mERβ2 activity. This data suggest that tissue responses to EDCs may not be easily predicted as they are dependent upon the levels of mERβ2 and ligand specificity. Our tissue-specific expression and *in vitro* data further provide evidence for a hypothesis that the negative regulation of mERβ1 and mERα by mERβ2 may play a regulatory physiological role by dampening tissue activity and toxic responses. A similar conclusion was proposed by Saijo et al. (2011), whose study suggested that mERβ2 may make tissues less sensitive to endogenous estrogen via this negative regulation. This may explain its role in the ovary where its expression at certain times would dampen the ERβ1 activity during folliculogenesis and deter untimely overstimulation. ERβ2 mRNA expression in the rat is increased in the mammary gland during lactation when the tissue is less responsive to estrogen (mERβ2 and rat ERβ2 have an 18 amino acid insert in the LBD and share 16 of 18 amino acids) (Lewandowski et al. 2002). Additionally, previous reports of dominant negative regulation of ERα and mERβ1 by mERβ2 suggest the relative levels of receptors within a tissue, not simply between tissues, may be an important factor for overall estrogenic signaling outcomes (Lu et al. 1998; Maruyama et al. 1998).

Co-regulators including co-activators and co-repressors interact with NRs such as ERs and other TFs to alter chromatin and stimulate or repress gene expression (Lonard and O’Malley 2012). Many co-regulators have been implicated in the physiology of reproduction, energy metabolism, inherited human genetic diseases, and cancer (Lonard and O’Malley 2012). SRC-1 was the first member of the p160 family of co-activators to be cloned, after which two additional family members, SRC-2 and SRC-3, were identified (Dasgupta and O’Malley 2014). Our previous studies demonstrated that both SRC2 and p300 co-activate ERα/ERE-mediated activity with BPA, BPAF, and Zea (Li et al. 2012). However, there is minimal information about mERβ coactivation with SRCs. From our current study, we conclude that all three SRC members have greater effect on mERβ1 responses than on mERβ2 responses in the presence of EDCs, and this co-activation occurs in a ligand-dependent manner. As expression of ER co-regulators is tissue specific, these findings could be informative in interpreting the tissue-specific and differential toxicities seen with EDCs especially from experimental animal toxicity studies where results are used to inform and contextualize human exposure studies and regulatory policies.

## Conclusions

The mERβ2 isoform, with 18 additional amino acids in the LBD, is expressed in several tissues including abundant expression in the ovary, lung, bladder, and colon. Like E_2_, a selection of EDCs failed to activate mERβ2 in our transactivation assay using HepG2 cells and an ERE luciferase reporter. We observed a decrease in ER transactivation activity when mERβ2 and mERβ1 were co-expressed compared with mERβ1 activity alone, suggesting the LBD structural alteration of mERβ2 confers a negative regulatory role in receptor-mediated estrogenic activity. Furthermore, mERβ2 exhibited reduced co-activation in the presence of EDCs, only selectively recruiting SRC2 or SRC3. Given the increasing use of experimental animal models to study the effects of EDCs, mERβ2 and its aberrant role in estrogen signaling should be considered when investigating mechanisms of toxicity. The potential for mERβ2 to attenuate tissue estrogenic activity in rodent models should be included when assessing human risk following EDC exposure, as there is no human homolog of this isoform and the activities it contributes would not be accounted for in the mechanistic application to human studies.

## Supplemental Material

(694 KB) PDF

## References

[r1] Antal MC, Petit-Demoulière B, Meziane H, Chambon P, Krust A (2012). Estrogen dependent activation function of ERβ is essential for the sexual behavior of mouse females.. Proc Natl Acad Sci U S A.

[r2] Berger A, Ziv-Gal A, Cudiamat J, Wang W, Zhou C, Flaws JA (2016). The effects of *in utero* bisphenol A exposure on the ovaries in multiple generations of mice.. Reprod Toxicol.

[r3] Berman T, Goldsmith R, Göen T, Spungen J, Novack L, Levine H (2013). Urinary concentrations of environmental contaminants and phytoestrogens in adults in Israel.. Environ Int.

[r4] Binder AK, Rodriguez KF, Hamilton KJ, Stockton PS, Reed CE, Korach KS (2013). The absence of ER-β results in altered gene expression in ovarian granulosa cells isolated from in vivo preovulatory follicles.. Endocrinology.

[r5] Burns KA, Li Y, Arao Y, Petrovich RM, Korach KS (2011). Selective mutations in estrogen receptor α D-domain alters nuclear translocation and non-estrogen response element gene regulatory mechanisms.. J Biol Chem.

[r6] Chu S, Fuller PJ (1997). Identification of a splice variant of the rat estrogen receptor β gene.. Mol Cell Endocrinol.

[r7] Couse JF, Yates MM, Deroo BJ, Korach KS (2005). Estrogen receptor-β is critical to granulosa cell differentiation and the ovulatory response to gonadotropins.. Endocrinology.

[r8] Dang ZC (2009). Dose-dependent effects of soy phyto-oestrogen genistein on adipocytes: mechanisms of action.. Obes Rev.

[r9] Dasgupta S, O’Malley BW (2014). Transcriptional coregulators: emerging roles of SRC family of coactivators in disease pathology.. J Mol Endocrinol.

[r10] Deroo BJ, Korach KS (2006). Estrogen receptors and human disease.. J Clin Invest.

[r11] Diamanti-Kandarakis E, Bourguignon JP, Giudice LC, Hauser R, Prins GS, Soto AM (2009). Endocrine-disrupting chemicals: an Endocrine Society scientific statement.. Endocr Rev.

[r12] Gaido KW, Leonard LS, Maness SC, Hall JM, McDonnell DP, Saville B (1999). Differential interaction of the methoxychlor metabolite 2,2-bis-(*p*-hydroxyphenyl)-1,1,1-trichloroethane with estrogen receptors α and β.. Endocrinology.

[r13] Hall JM, McDonnell DP (2005). Coregulators in nuclear estrogen receptor action: from concept to therapeutic targeting.. Mol Interv.

[r14] Hanstein B, Liu H, Yancisin MC, Brown M (1999). Functional analysis of a novel estrogen receptor- β isoform.. Mol Endocrinol.

[r15] Hartman J, Ström A, Gustafsson JÅ (2012). Current concepts and significance of estrogen receptor β in prostate cancer.. Steroids.

[r16] Henley DV, Korach KS (2010). Physiological effects and mechanisms of action of endocrine disrupting chemicals that alter estrogen signaling.. Hormones (Athens).

[r17] Hormann AM vom Saal FS Nagel SC Stahlhut RW Moyer CL Ellersieck MR 2014 Holding thermal receipt paper and eating food after using hand sanitizer results in high serum bioactive and urine total levels of bisphenol A (BPA). PLoS One 9 e110509, doi:10.1371/journal.pone.0110509 25337790 PMC4206219

[r18] Jefferson WN, Patisaul HB, Williams CJ (2012). Reproductive consequences of developmental phytoestrogen exposure.. Reproduction.

[r19] Kuiper GG, Carlsson B, Grandien K, Enmark E, Häggblad J, Nilsson S (1997). Comparison of the ligand binding specificity and transcript tissue distribution of estrogen receptors α and β.. Endocrinology.

[r20] Le HH, Belcher SM (2010). Rapid signaling actions of environmental estrogens in developing granule cell neurons are mediated by estrogen receptor β.. Endocrinology.

[r21] Lewandowski S, Kalita K, Kaczmarek L (2002). Estrogen receptor β. Potential functional significance of a variety of mRNA isoforms.. FEBS Lett.

[r22] Li Y Burns KA Arao Y Luh CJ Korach KS 2012 Differential estrogenic actions of endocrine-disrupting chemicals bisphenol A, bisphenol AF, and zearalenone through estrogen receptor α and β *in vitro*. Environ Health Perspect 120 1029 1035, doi:10.1289/ehp.1104689 22494775 PMC3404668

[r23] Li Y Luh CJ Burns KA Arao Y Jiang Z Teng CT 2013 Endocrine-disrupting chemicals (EDCs): *in vitro* mechanism of estrogenic activation and differential effects on ER target genes. Environ Health Perspect 121 459 466, doi:10.1289/ehp.1205951 23384675 PMC3620735

[r24] Lonard DM, O’Malley BW (2007). Nuclear receptor coregulators: judges, juries, and executioners of cellular regulation.. Mol Cell.

[r25] Lonard DM, O’Malley BW (2012). Nuclear receptor coregulators: modulators of pathology and therapeutic targets.. Nat Rev Endocrinol.

[r26] Lu B, Leygue E, Dotzlaw H, Murphy LJ, Murphy LC, Watson PH (1998). Estrogen receptor-β mRNA variants in human and murine tissues.. Mol Cell Endocrinol.

[r27] Maruyama K, Endoh H, Sasaki-Iwaoka H, Kanou H, Shimaya E, Hashimoto S (1998). A novel isoform of rat estrogen receptor beta with 18 amino acid insertion in the ligand binding domain as a putative dominant negative regular of estrogen action.. Biochem Biophys Res Commun.

[r28] McDonnell DP, Norris JD (2002). Connections and regulation of the human estrogen receptor.. Science.

[r29] Miller KK, Al-Rayyan N, Ivanova MM, Mattingly KA, Ripp SL, Klinge CM (2013). DHEA metabolites activate estrogen receptors alpha and beta.. Steroids.

[r30] Nilsson S, Mäkelä S, Treuter E, Tujague M, Thomsen J, Andersson G (2001). Mechanisms of estrogen action.. Physiol Rev.

[r31] O’Lone R, Frith MC, Karlsson EK, Hansen U (2004). Genomic targets of nuclear estrogen receptors.. Mol Endocrinol.

[r32] Patel S Zhou C Rattan S Flaws JA 2015 Effects of endocrine-disrupting chemicals on the ovary. Biol Reprod 93 20, doi:10.1095/biolreprod.115.130336 26063868 PMC6366440

[r33] Pellegrini M, Bulzomi P, Lecis M, Leone S, Campesi I, Franconi F (2014). Endocrine disruptors differently influence estrogen receptor β and androgen receptor in male and female rat VSMC.. J Cell Physiol.

[r34] Petersen DN, Tkalcevic GT, Koza-Taylor PH, Turi TG, Brown TA (1998). Identification of estrogen receptor β2, a functional variant of estrogen receptor β expressed in normal rat tissues.. Endocrinology.

[r35] Rodriguez KF, Couse JF, Jayes FL, Hamilton KJ, Burns KA, Taniguchi F (2010). Insufficient luteinizing hormone-induced intracellular signaling disrupts ovulation in preovulatory follicles lacking estrogen receptor-β.. Endocrinology.

[r36] Saijo K, Collier JG, Li AC, Katzenellenbogen JA, Glass CK (2011). An ADIOL-ERβ-CtBP transrepression pathway negatively regulates microglia-mediated inflammation.. Cell.

[r37] Shelnutt S, Kind J, Allaben W (2013). Bisphenol A: update on newly developed data and how they address NTP’s 2008 finding of “Some Concern.”. Food Chem Toxicol.

[r38] Sobolewski M, Conrad K, Allen JL, Weston H, Martin K, Lawrence BP (2014). Sex-specific enhanced behavioral toxicity induced by maternal exposure to a mixture of low dose endocrine-disrupting chemicals.. Neurotoxicology.

[r39] Tsai MJ, O’Malley BW (1994). Molecular mechanisms of action of steroid/thyroid receptor superfamily members.. Annu Rev Biochem.

[r40] Wetherill YB, Akingbemi BT, Kanno J, McLachlan JA, Nadal A, Sonnenschein C (2007). *In vitro* molecular mechanisms of bisphenol A action.. Reprod Toxicol.

[r41] Zhao C, Toresson G, Xu L, Koehler KF, Gustafsson JA, Dahlman-Wright K (2005). Mouse estrogen receptor β isoforms exhibit differences in ligand selectivity and coactivator recruitment.. Biochemistry.

