# Supplementary material for: Differential Activation of a Mouse Estrogen Receptor β Isoform (mERβ2) with Endocrine-Disrupting Chemicals (EDCs)
Source: Environ Health Perspect. 2016 Sep 16;125(4):634–42. doi: 10.1289/EHP396 (PMC5381991; doi:10.1289/EHP396)
Supplement: (694 KB) PDF [file EHP396.s001.acco.pdf]

Environ Health Perspect
DOI: 10.1289/EHP396

Note to readers with disabilities: *EHP* strives to ensure that all journal content is accessible to all readers. However, some figures and Supplemental Material published in *EHP* articles may not conform to 508 standards due to the complexity of the information being presented. If you need assistance accessing journal content, please contact ehponline@niehs.nih.gov. Our staff will work with you to assess and meet your accessibility needs within 3 working days.

Supplemental Material

Differential Activation of a Mouse Estrogen Receptor β Isoform (mERβ2) with Endocrine-Disrupting Chemicals (EDCs)

Lauren J. Donoghue, Thomas I. Neufeld, Yin Li, Yukitomo Arao, Laurel A. Coons, and Kenneth S. Korach

Table of Contests

Table S1: The chemical structures of the compounds used in this study.
Table S2: The hormones and Endocrine disrupting chemicals (EDCs) used in this study
Table S3: Plasmid amounts for transfection experiments by figure
Figure S1: HepG2 cells were transfected with the 3xERE-luc reporter plasmid, pRL-TK transfection plasmid and the expression plasmid for mERα, mERβ2 or both mERα and mERβ2. Cells recover for 18 hr and were then treated with 10^{-8} M E_2 (mERα), 10^{-7} M E_2 (mERβ2) or other compounds for 18 hr. The luciferase activity is represented as relative activity compared with the vehicle treated cells transfected with empty pcDNA3 plasmid. The relative activity is represented as the mean ± SEM. Assays were run in triplicate and data replicated over at least three independent experiments.
Figure S2: HepG2 cells were transfected with the 3xERE-luc reporter plasmid, pRL-TK plasmid and the expression plasmid for mERα, mERβ2 or both mERα and mERβ2 with or without SRC2 expression plasmid (see detail in Table S4). Cells recovered for 18 hr and then treated with increasing doses (10^{-8} to 10^{-6} M) of compounds for 18 hr. The luciferase activity is represented as relative activity compared with the vehicle treated cells transfected with empty pcDNA3 plasmid. The relative activity is represented as the mean ± SEM. Assays were run in triplicate and data replicated over at least three independent experiments.
Table S4: Plasmid amounts for transfection experiments

Environ Health Perspect
DOI: 10.1289/EHP396

Table S1. The chemical structures of the compounds used in this study

Hormones and pharmaceutical drugs

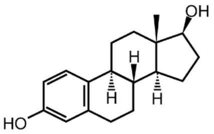
17β-estradiol (E2)

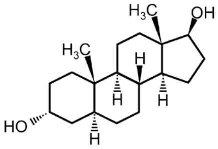
5α-Androstane-3β, 17β-diol (3β-diol)

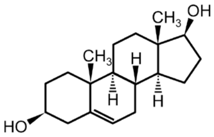
Androst-5-ene-3β, 17β-diol (Δ5-diol)

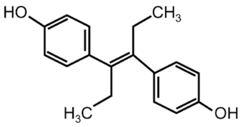
Diethylstilbestrol (DES)

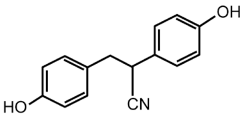
Diarylpropionitrile (DPN)

Group 1

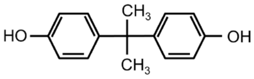
Bisphenol A (BPA)

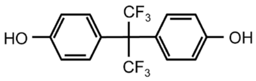
Bisphenol AF (BPAF)

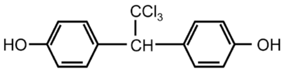
2-2-bis(p-hydroxyphenyl)-1,1,1-trichloroethane (HPTE)

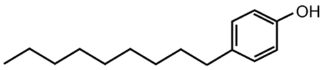
4-n-nonylphenol (4n-NP)

Group 2

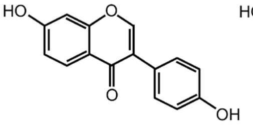
Daidzein (Dai)

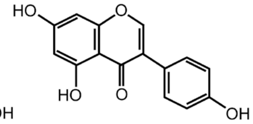
Genistein (Gen)

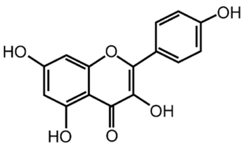
Kaempferol (Kaem)

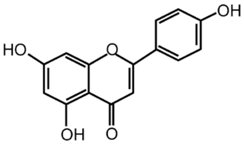
Apigenin (Api)

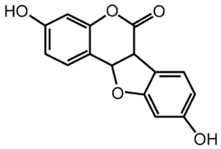
Coumestrol (Coum)

Group 3

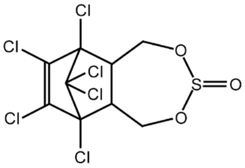
Endosulfan (Endo)

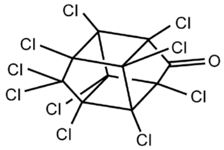
Kepone (Kep)

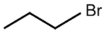
1-Bromopropane (1-BP)

Table S2: The hormones and Endocrine disrupting chemicals (EDCs) used in this study

Compounds	CAS No.	Chemical class	Product class	MW
17β-estradiol (E_2)	50-28-2	Steroid, Phenolic; Estrene	Hormone	272.38
5α-Androstane-3β,17β -diol (3β-diol)	571-20-0	Steroid, Phenolic	Hormone	292.46
androst-5-ene-3β,17β -diol (Δ^5-diol)	521-17-5	Steroid, Phenolic	Hormone	290.44
Diethylstilbestrol (DES)	56-53-1	Phenolic; Nonsteroidal Estrogen	Pharmaceutical	268.35
Diarylpropionitrile (DPN)	1428-67-7	Phenolic, Nonsteroidal Estrogen	Pharmaceutical	239.27
**Group 1**				
Bisphenol A (BPA)	80-05-7	Diphenylalkane; Bisphenol; Phenol	Chemical intermediate	228.29
Bisphenol AF (BPAF)	1478-61-1	Diphenylalkane; Bisphenol; Phenol	Chemical intermediate	336.23
2-2-bis(p-hydroxyphenyl)-1,1,1-trichloroethane (HPTE)	72-43-5	Diphenylalkane; Bisphenol; Phenol	Chemical intermediate	317.59
4-n-Nonylphenol (4-n-NP)	104-40-5	Alkylphenol; Phenol	Chemical intermediate	220.35
**Group 2**				
Daidzein (Dai)	486-66-8	Flavanoid; Isoflavone; Phenol	Natural product	254.23
Genistein (Gen)	446-72-0	Flavanoid; Isoflavone; Phenol	Natural product	270.24
Kaempferol (Kaem)	520-18-3	Flavanoid; Isoflavone ; Phenol	Natural product	286.23
Apigenin (Api)	520-36-5	Flavanoid; Flavones ; Phenol	Natural product	270.24
Coumestrol (Coum)	479-13-0	Flavanoid; Isoflavone; Phenol	Natural product	282.22
**Group 3**				
Endosulfan (Endo)	115-29-7	Organochlorine	Pesticide	406.93
Kepone (Kep)	143-50-0	Organochlorine	Pesticide	490.64
1-Bromopropane (1-BP)	106-94-5	Organobromine	Chemical intermediate	122.99

Table S3. Plasmid amounts for transfection experiments by Figure

	Total DNA (µg)	mERβ2 (µg)	mERβ1 (µg)	Vector (µg)	SRC (µg)	3xERE-luc (µg)	pRL-TK (µg)
**Figure 2 (screening)**							
mERβ2	0.5	0.1		0.1		0.2	0.1
mERβ1	0.5		0.1	0.1		0.2	0.1
**Figure 3 (dose curves)**							
mERβ2	0.5	0.1		0.1		0.2	0.1
mERβ1	0.5		0.1	0.1		0.2	0.1
mERβ2+mERβ1	0.5	0.1	0.1			0.2	0.1
**Figure 4 (SRC+)**							
mERβ2	0.7	0.1		0.3		0.2	0.1
mERβ2+SRC	0.7	0.1			0.3	0.2	0.1
mERβ1	0.7		0.1	0.3		0.2	0.1
mERβ1+SRC	0.7		0.1		0.3	0.2	0.1

Figure S1

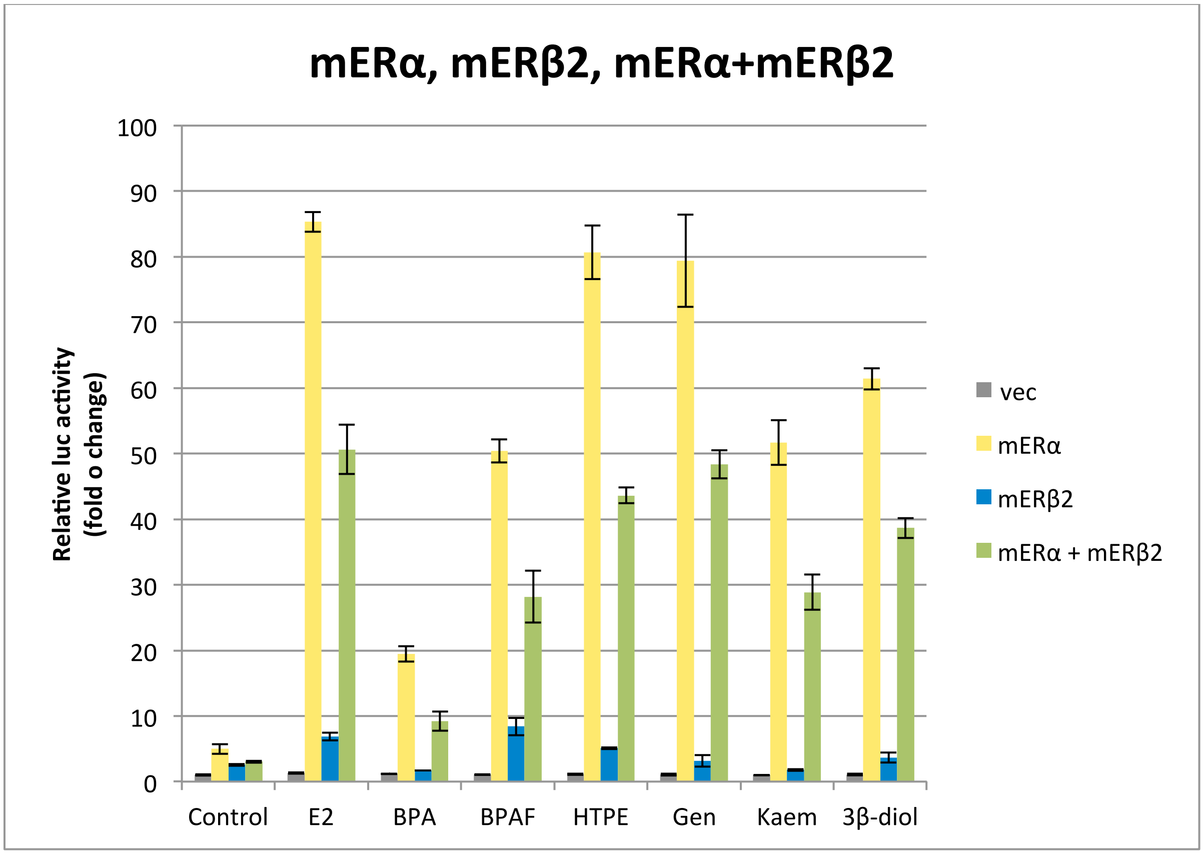

Figure S1. HepG2 cells were transfected with the 3xERE-luc reporter plasmid, pRL-TK transfection plasmid and the expression plasmid for mERα, mERβ2 or both mERα and mERβ2. Cells recover for 18 hr and were then treated with 10^{-8} M E_2 (mERα), 10^{-7} M E_2 (mERβ2) or other compounds for 18 hr. The luciferase activity is represented as relative activity compared with the vehicle treated cells transfected with empty pcDNA3 plasmid. The relative activity is represented as the mean ± SEM. Assays were run in triplicate and data replicated over at least three independent experiments.

Figure S2

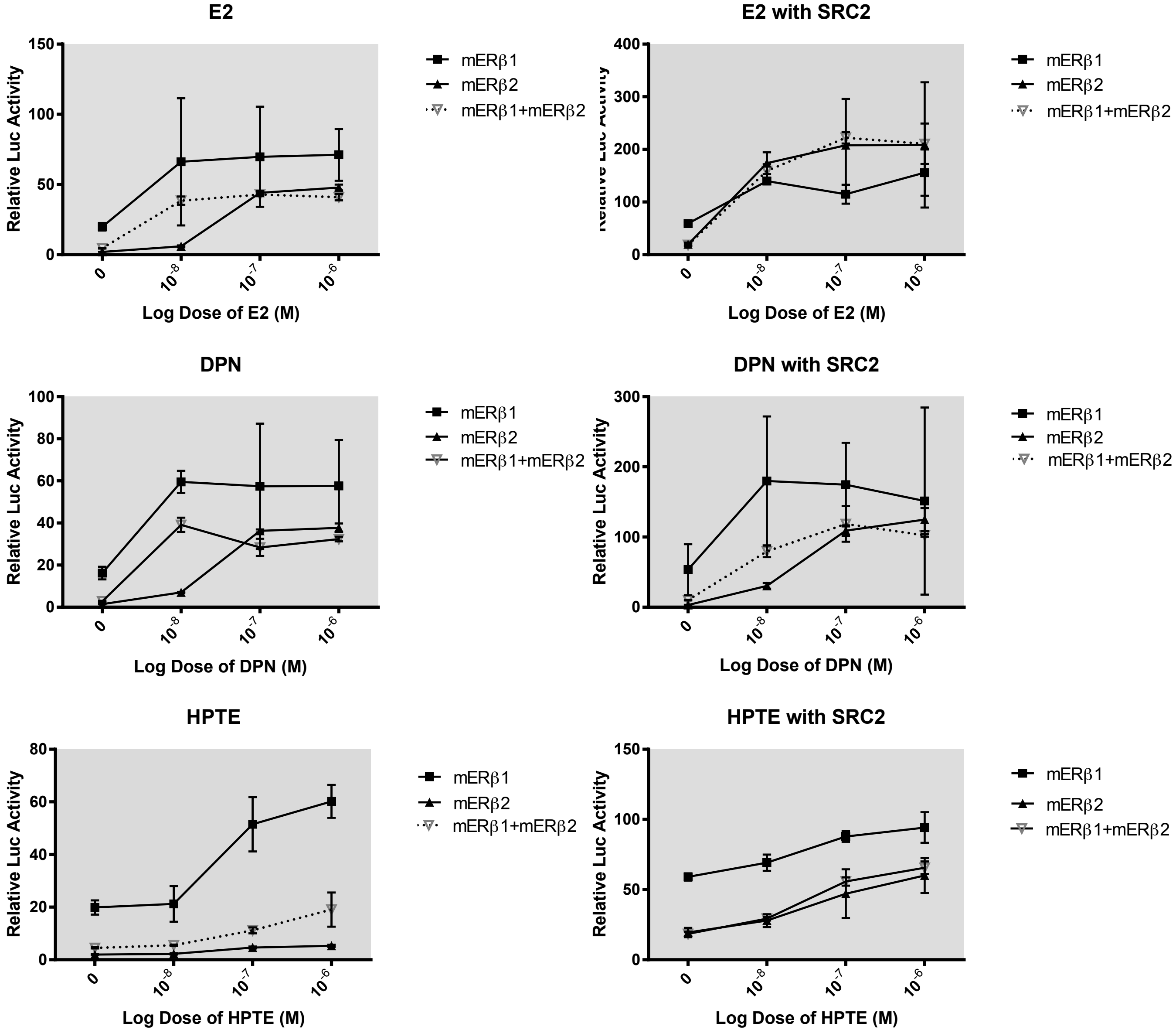

Figure S2. HepG2 cells were transfected with the 3xERE-luc reporter plasmid, pRL-TK plasmid and the expression plasmid for mERα, mERβ2 or both mERα and mERβ2 with or without SRC2 expression plasmid (see detail in Table S4). Cells recovered for 18 hr and then treated with increasing doses (10^{-8} to 10^{-6} M) of compounds for 18 hr. The luciferase activity is represented as relative activity compared with the vehicle treated cells transfected with empty pcDNA3 plasmid. The relative activity is represented as the mean ± SEM. Assays were run in triplicate and data replicated over at least three independent experiments.

Table S4. Plasmid amounts for transfection experiments

Figure S2: E2, DPN and HPTE dose curve (mERβ isoforms + SRC2)							
	Total DNA (μg)	mERβ2 (μg)	mERβ1 (μg)	Vector (μg)	SRC2 (μg)	3xERE-luc (μg)	pRL-TK (μg)
mERβ2	0.8	0.1		0.4		0.2	0.1
mERβ2+SRC	0.8	0.1		0.1	0.3	0.2	0.1
mERβ1	0.8		0.1	0.4		0.2	0.1
mERβ1+SRC	0.8		0.1	0.1	0.3	0.2	0.1
mERβ2+mERβ1	0.8	0.1	0.1	0.3		0.2	0.1
mERβ2+mERβ1+SRC2	0.8	0.1	0.1		0.3	0.2	0.1
